# AKAP1 Regulates Mitochondrial Dynamics during the Fatty-Acid-Promoted Maturation of Human-Induced Pluripotent Stem Cell-Derived Cardiomyocytes as Indicated by Proteomics Sequencing

**DOI:** 10.3390/ijms24098112

**Published:** 2023-04-30

**Authors:** Han Xiang, Hao Xu, Bin Tan, Qin Yi, Xinyuan Zhang, Rui Wang, Tangtian Chen, Qiumin Xie, Jie Tian, Jing Zhu

**Affiliations:** 1 Department of Pediatric Research Institute, National Clinical Research Center for Child Health and Disorders, Ministry of Education Key Laboratory of Child Development and Disorders, Chongqing Key Laboratory of Pediatrics, China International Science and Technology Cooperation Base of Child Development and Critical Disorders, Children’s Hospital of Chongqing Medical University, Chongqing 400014, China; 2 Department of Clinical Laboratory, Children’s Hospital of Chongqing Medical University, Chongqing 400014, China; 3 Department of Clinical Laboratory, Women and Children’s Hospital of Chongqing Medical University, Chongqing 400016, China; 4 Department of Cardiovascular (Internal Medicine), Children’s Hospital of Chongqing Medical University, Chongqing 400014, China

**Keywords:** HiPSC-CM, fatty acid, AKAP1, mitochondrial fusion

## Abstract

Human-induced pluripotent stem cell-derived cardiomyocytes (hiPSC-CMs) are cells with promising applications. However, their immaturity has restricted their use in cell therapy, disease modeling, and other studies. Therefore, the current study focused on inducing the maturation of CMs. We supplemented hiPSC-CMs with fatty acids (FAs) to promote their phenotypic maturity. Proteomic sequencing was performed to identify regulators critical for promoting the maturation of hiPSC-CMs. AKAP1 was found to be significantly increased in FA-treated hiPSC-CMs, and the results were verified. Therefore, we inhibited AKAP1 expression in the FA-treated cells and analyzed the outcomes. FA supplementation promoted the morphological and functional maturation of the hiPSC-CMs, which was accompanied by the development of a mitochondrial network. Proteomic analysis results revealed that AKAP1 expression was significantly higher in FA-treated hiPSC-CMs than in control cells. In addition, increased phosphorylation of the mitochondrial dynamin Drp1 and an increased mitochondrial fusion rate were found in FA-treated hiPSC-CMs. After AKAP1 was knocked down, the level of DRP1 phosphorylation in the cell was decreased, and the mitochondrial fusion rate was reduced. FA supplementation effectively promoted the maturation of hiPSC-CMs, and in these cells, AKAP1 regulated mitochondrial dynamics, possibly playing a significant role.

## 1. Introduction

Cardiovascular diseases are the primary reasons for human illness and mortality [1]. Pluripotent stem cell (PSC) technology [2,3,4] that allows cardiovascular cells to be acquired from patients and healthy individuals, in some instances, genetically matched through mutational repair, may lead to a paradigm shift in human cardiovascular disease research [5,6,7]. Although human-induced pluripotent stem cell-derived cardiomyocytes (hiPSC-CMs) have shown great potential for cardiac disease modeling and autologous tissue transplantation, their immaturity has largely limited their applications [2].

In recent years, researchers have performed substantial research on the maturity of hiPSC-CMs [8,9,10]. Supplementation of cell culture medium with fatty acids (FAs) is a classic method to promote myocardial maturation [11]. After delivery, as an infant transitions from placental feeding to drinking breast milk, a significant metabolic shift, from glycolysis to FA β-oxidation for ATP generation, is observed. However, glucose in standard cell culture medium (RPMI-B27-insulin) is the primarily source of cellular energy and contains very little lipid (10 mM total lipid, much below the serum FA content of 300 mM in human babies). Therefore, in the current study, the addition of free FA to the culture media was utilized to increase the maturity of hiPSC-CMs [12,13,14,15]. Nonetheless, identification of the underlying mechanism requires further study.

Mitochondria are essential regulators of cardiac development and differentiation, as well as coordinators of cellular energy and metabolism [16,17]. Increasing evidence has shown that mitochondria in mature CMs develop highly dynamic networks, which are maintained mainly via equilibrating mitochondrial fusion and fission [18]. Extended (>100 days) periods of hiPSC-CMs in vitro culture contribute to the maturation of the heart cells, in which enhanced mitochondrial biogenesis, membrane potential, and sophisticated morphological and functional mitochondrial networks have been observed [19]. In addition, mitochondrial lengthening and dynamic network development have been observed after culture medium supplementation with FA [12,15], but the precise mechanisms underlying FA effects still need to be determined. Therefore, to obtain a greater knowledge of the processes and pathways that promote the maturity of hiPSC-CMs, we performed proteome research and analyses on hiPSC-CMs treated with or without FA.

Mitochondrial A-kinase anchor protein1 (AKAP1), also named AKAP121 or human homolog AKAP149, is a scaffold protein in the AKAP family. AKAP1 has been shown to be a key factor regulating mitochondrial functions [20,21]. Akap1-deficient mice develop dysfunctional mitochondria [22]. Increasing research reports have indicated that variations in AKAP1 levels, which may modulate the phosphorylation of DRP1, influence mitochondrial dynamics [23]. However, the regulatory role of AKAP1 in mitochondrial dynamics in hiPSC-CMs has not been explored to date.

In our experiments, a developed mitochondrial network was observed in FA-treated hiPSC-CMs, and AKAP1 expression was significantly higher in the FA group than in the control group, as determined by proteomic sequencing. Hence, we hypothesized that AKAP1 may regulate mitochondrial function to promote the maturation of FA-treated hiPSC-CMs. We are the first to have focused on AKAP1, which was significantly elevated in FA-treated hiPSC-CMs, as determined by proteomics, and to have detected a role for AKAP1 in hiPSC-CMs maturation; namely, the regulation of mitochondrial function.

## 2. Results

### 2.1. Generation and Characterization of hiPSC-CMs

**Figure 1:**
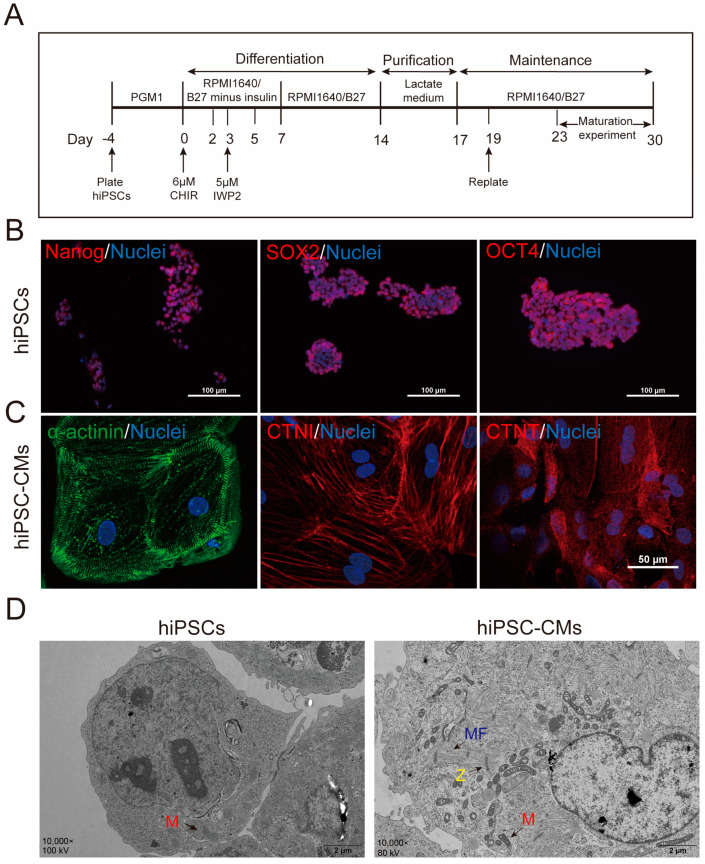
Characterization and identification of hiPSCs and hiPSC-CMs. (**A**) Diagram showing the method of hiPSCs differentiation into cardiomyocytes (hiPSC-CMs) and maturation process of hiPSC-CMs; PGM1 and RMPI: cell culture medium; B27: medium supplement; CHIR: a highly selective inhibitor of GSK3; IWP2: inhibitor of Wnt/β-catenin signaling. (**B**) HiPSCs grew in a “colony-like” pattern expressing the pluripotent stem cell-specific markers Nanog, SOX2, and OCT4. Blue is the nucleus stained with Hoechst 33342. Scale bar = 100 µm. (**C**) HiPSC-CMs were reinoculated to grow as monolayers and express the cardiomyocyte-specific markers myosin α-actinin (green), CTNI (red), and CTnT (red). (**D**) Electron microscopy results of hiPSC-CMs in B27 and FA groups, TEM images of hiPSC-CMs; M: mitochondria (red arrows); MF: myogenic fibers (blue arrows); Z: Z-line (yellow arrows).

HiPSCs are cultured as shown in Figure 1A. HiPSCs grow in a “colony-like” pattern and express the pluripotent stem cell-specific markers Nanog, SOX2, and OCT4 (Figure 1B). HiPSC-CMs begin spontaneous contraction after 7 days of differentiation, with enhanced contraction occurring between 7 and 10 days (Video S1). HiPSC-CMs grew into monolayers after 3 days of purification and reinoculation on day 18 (Video S2). The purified cells expressed cardiac-specific markers, such as CTNI, α-actinin, and CTNT (Figure 1C). Electron microscopic examination of hiPSCs and hiPSC-CMs after induction revealed that hiPSC-CMs showed myocardial-specific structures such as myofilaments (MF) and myosin (Z-line), as well as significant changes in mitochondrial morphology and structure. HiPSCs had fewer mitochondria and fewer mitochondrial cristae, whereas hiPSC-CMs had significantly more mitochondria and more dense mitochondrial cristae (Figure 1D).

### 2.2. Proteomic Sequencing

**Figure 2:**
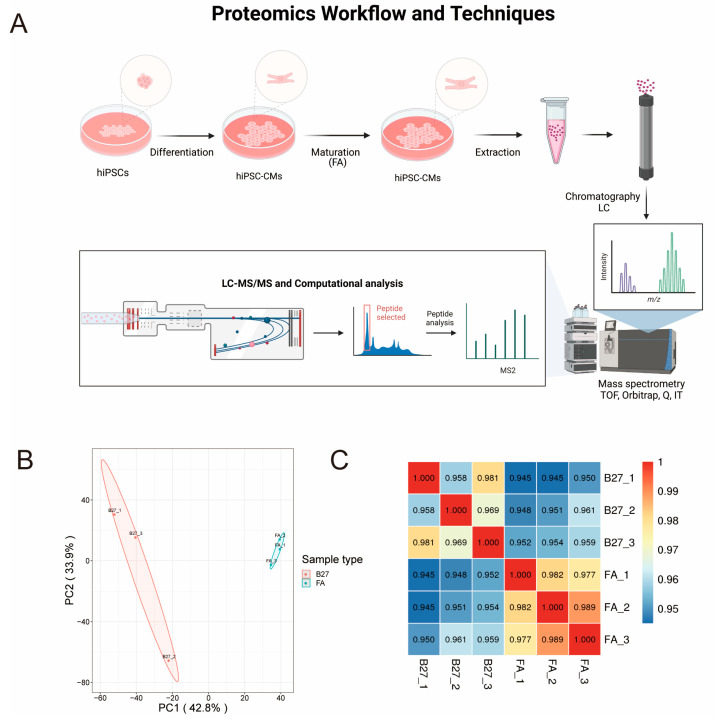
Proteomics workflow and protein expression. (**A**) Proteomics workflow and techniques. (**B**) PCA analysis of the hiPSC-CMs. (**C**). Correlation heap map of hiPSC-CMs samples. Numbers in boxes represent Pearson’s correlation coefficient between two corresponding samples.

To better understand the molecular mechanisms involved in the maturation of hiPSC-CMs, we performed proteomic sequencing of hiPSC-CMs treated with maturation medium (fatty acid supplementation in the RPM1/B27 medium) (Figure 2A); to establish a control group, we collected three groups of hiPSC-CMs treated with normal medium (RPM1/B27 medium). We needed to determine whether the quantitative results were statistically consistent in biological and technical replicates. The reproducibility was evaluated using Pearson’s correlation coefficient (PCC) analysis and principal component analysis (PCA). Quantitative PCA of proteins from hiPSC-CMs treated with maturity-inducing or normal medium revealed a substantial degree of sample clustering, indicating a high degree of sample variability (Figure 2B). A heat map was based on PCCs between pairs of all samples (Figure 2C). The coefficient was used to measure the linear correlation between the two data sets: a PCC close to −1 indicated a negative correlation, a PCC close to 1 indicated a positive correlation, and a PCC close to 0 indicated no correlation.

**Figure 3:**
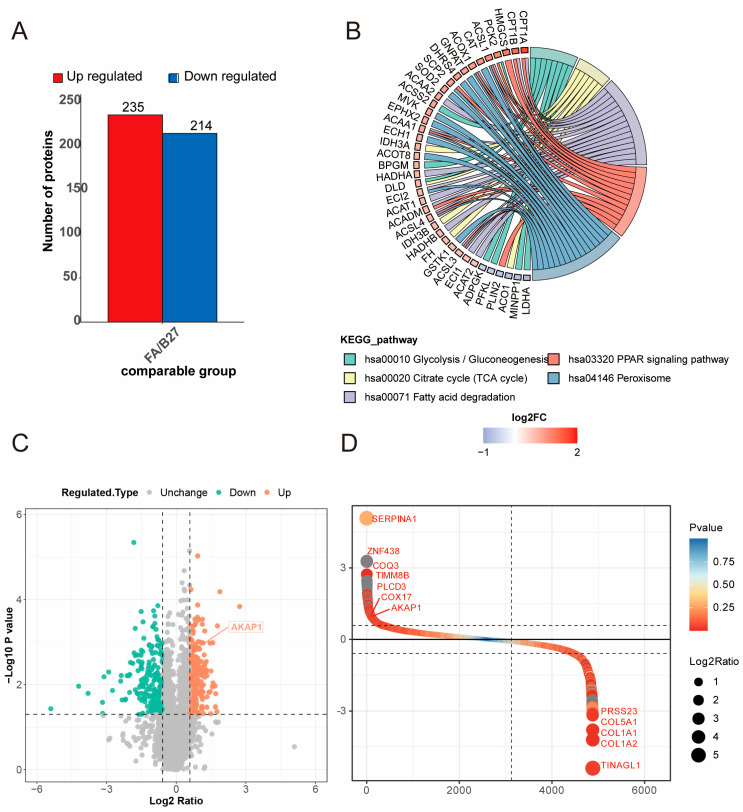
Proteins expression of hiPSC-CMs in B27 and FA groups. (**A**) Differentially expressed protein numbers of hiPSC-CMs between B27 and FA groups. (**B**) Representative significantly enriched KEGG pathways. Adjusted *p* value (FDR) < 0.5 was considered significant in KEGG analysis. (**C**) Volcano plot of the differential expression proteins. (**D**) Fold change ranking of the differential expression proteins.

We identified 449 differentially expressed genes (DEGs), of which 235 were upregulated genes and 214 downregulated genes in FA-treated hiPSC-CMs (Figure 3A). The abovementioned DEGs were compared using GO and KEGG enrichment analyses (Figure 3B). The results revealed that in FA-treated cells, DEGs were enriched in several signaling pathways. The enriched pathways for upregulated genes were PPAR signaling pathway (CPT1A, CPT1B, ACSL1, etc.), TCA cycle (IDH3A, IDH3B, etc.), peroxisome (ACOX1, PCK2, CPT1, ECH, etc.), fatty acid degradation (ACAT1, ACADM, etc.); down-regulated gene enriched pathways include glycolysis (LDHA, PFKL). Among the upregulated DEGs (orange) and downregulated proteins (cyan), we discovered that the expression of AKAP1 was significantly elevated (Figure 3C,D). Therefore, we further investigated the role of AKAP1 in promoting the maturation of hiPSC-CMs treated with FA.

### 2.3. Fatty Acids Promote the Morphological and Structural Maturation of hiPSC-CMs

The total FA concentration in human neonates is approximately 300 μM, of which palmitic acid accounts for approximately 34%, oleic acid accounts for approximately 27%, and linoleic acid accounts for 15%. Therefore, in this study, to simulate the FA environment during development in vivo, we treated hiPSC-CMs with equal proportions of palmitic acid albumin, oleic acid albumin, and linoleic acid albumin complexes. We added 115.5 M FA to RPMI-B27-insulin medium (glucose-free) following reports in the literature suggesting that one million cells consume approximately 57 ± 7 mM FA in two days [13].

**Figure 4:**
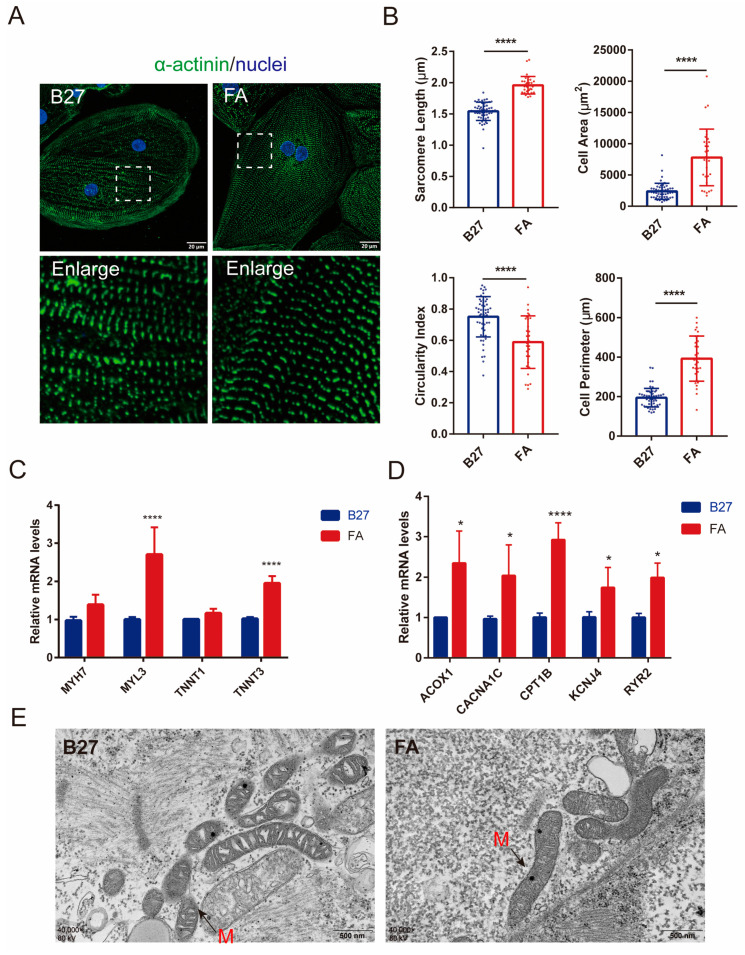
Fatty acids promote the structural maturation of hiPSC-CMs. (**A**) α-actinin (green) and Hoechst (blue) staining. Scale bar = 20 μm. (**B**) Cell perimeter, cell area, and sarcomere length of hiPSC-CMs in the fatty acid group were significantly increased compared to the B27 group, while the circularity index of cells referred to significantly decreased. (*n* = 56 cells for B27 and *n* = 33 cells for FA, from 3 independent experiments); **** *p* < 0.0001. (**C**,**D**) Real-time PCR analysis of maturation-related gene expression in FA-treated hiPSC-CMs. *n* = 4. * *p* ˂ 0.05, **** *p* ˂ 0.0001. (**E**) Electron microscopy results of hiPSC-CMs in B27 and FA groups, TEM images of hiPSC-CMs; M: mitochondria (red arrows).

To examine the effect of FA on hiPSC-CMs, we stained for α-actinin and found that compared with the B27 group, cell perimeter, cell area, and monomer length were significantly increased, while the cell roundness index [4π area/(perimeter)^2^] (where “0 “ denotes the theoretical minimum of ideal rod-shaped cells, and “1” denotes ideal round cells) was decreased (Figure 4A,B). Q-PCR was performed to examine myocardial structure, FA metabolism, and ion channel-related gene expression in the FA and B27 groups (Figure 4C,D). Significant increases in myosin heavy chain (MYH)7, myosin light chain (MYL)3, TNNI1, and TNNI3 levels were observed in hiPSC-CMs, which was consistent with reports suggesting that CM maturation is accompanied by a shift from embryonic TNNI1 expression to postnatal TNNI3 expression. In addition, the FA group showed significantly elevated levels of calcium voltage-gated channel subunit 1C (CACNA1C), ryanodine receptor 2 (RYR2), potassium voltage-gated channel subfamily J (KCNJ)4, and genes encoding -FA oxidation (CPT1B and ACOX1). The results indicated that the morphological structure of hiPSC-CMs was more developed in the FA group than in the B27 group.

### 2.4. Fatty Acids Promote Mitochondrial Structure and Respiratory Reserve Capacity in hiPSC-CMs

**Figure 5:**
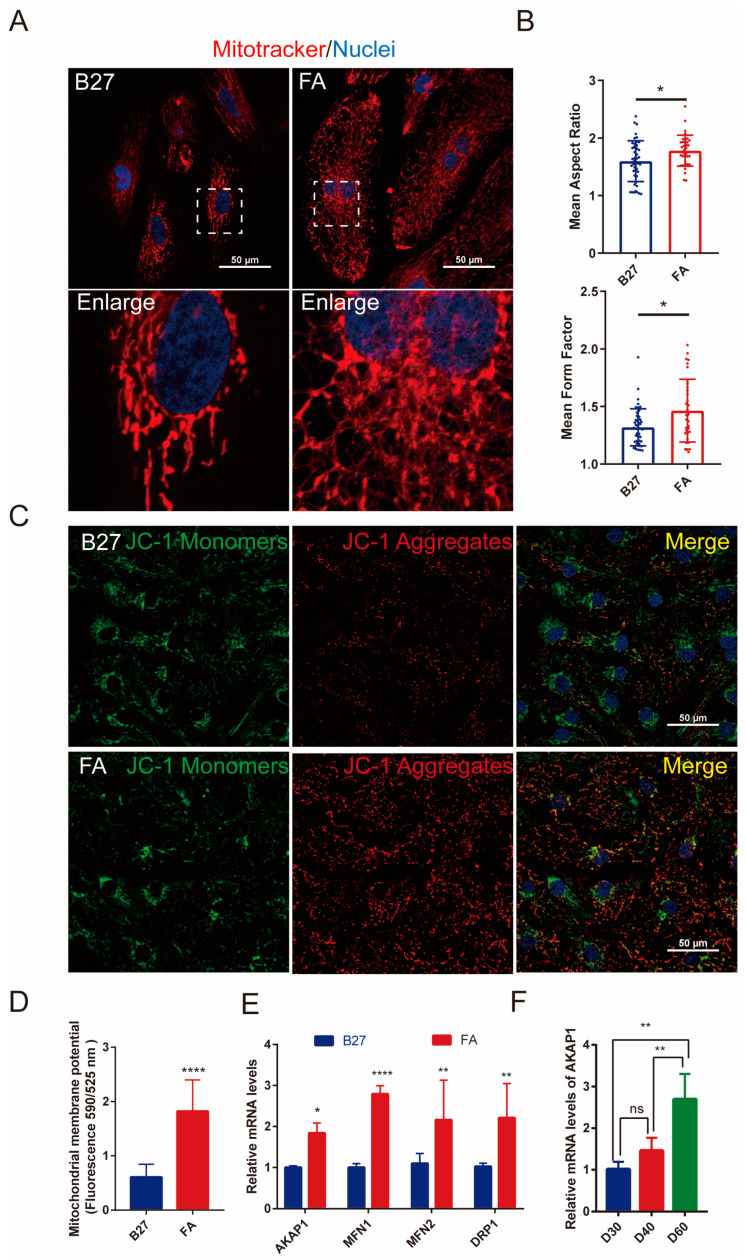
Mitochondrial morphology of FA-treated hiPSC-CMs. (**A**) MitoTracker staining of mitochondria (red) and nuclei (blue), scale bar = 50 μm. (**B**) MitoTracker staining statistics, the average morphological factor of cell mitochondria and average aspect ratio of mitochondria. (*n* = 46 cells for B27 and *n* = 35 cells for FA, from 3 independent experiments). * *p* ˂ 0.05. (**C**) JC-1 assay for mitochondrial membrane potential expression. Scale bar = 50 μm. JC-1 monomer (red fluorescence), JC-1 polymer (green); (**D**) mitochondrial potential red-green fluorescence ratio. *n* = 3. **** *p* ˂ 0.0001. (**E**) Real-time PCR analysis of mitochondria dynamin-related gene expression in FA-treated hiPSC-CMs. *n* = 3. * *p* ˂ 0.05, ** *p* ˂ 0.01, **** *p* ˂ 0.0001. (**F**) Real-time PCR analysis of AKAP1 gene expression in hiPSC-CMs on D30, D40, and D60. *n* = 3. ** *p* ˂ 0.01. ns=not statistically significant.

In the FA treatment group, electron microscopy showed longer mitochondria with denser mitochondrial cristae in hiPSC-CMs compared to the B27 group (Figure 4E). MitoTracker staining revealed significant changes in mitochondrial morphology (Figure 5A,B). Compared to the B27 group, the FA treatment group exhibited a more abundant filamentous mitochondrial network (Figure 5A). The mean form factor and mean aspect ratio of cellular mitochondria were significantly increased (*p* < 0.05), suggesting growth in mitochondrial morphology and increased enrichment (Figure 5B). Another alteration is increased mitochondrial membrane potential (MMP or ΔΨm) in hiPSC-CMs after FA treatment, as measured by JC-1 staining, suggesting higher mitochondrial functionality (Figure 5C,D). The increased expression of the mitochondrial dynamics-related genes AKAP1, MFN1, MFN2, and p-DRP1 after FA treatment suggested that the mitochondrial fusion rate was increased (Figure 5E). In addition, we performed Q-PCR to determine AKAP1 gene expression in hiPSC-CMs on D30, D40, and D60 and discovered that AKAP1 expression gradually increased with time (Figure 5F).

**Figure 6:**
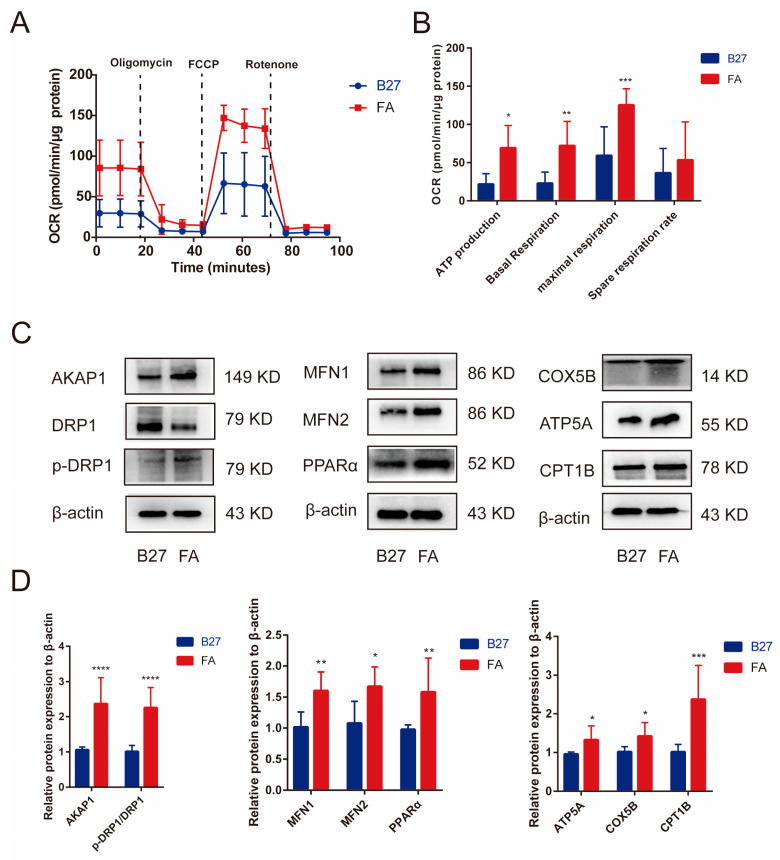
Mitochondrial respiration function and mitochondrial dynamics analysis. (**A**) Representative fatty acid oxidation of B27 and FA in hiPSC-CMs after incubation with the oligomycin, FCCP, and rotenone. (**B**) Quantification of maximum maximal respiratory capacity, ATP production, and spare respiratory capacity of hiPSC-CMs. *n* = 3; * *p* ˂ 0.05, ** *p* ˂ 0.01, *** *p* ˂ 0.001. (**C**,**D**) Western blot analysis of relative expression levels of AKAP1, Drp1, p-Drp1, MFN1, MFN2, PPARα, ATP5A, COX5B, and CPT1B in hiPSC-CMs between FA group and B27 group. *n* > 3. * *p* ˂ 0.05, ** *p* ˂ 0.01, *** *p* ˂ 0.001, **** *p* ˂ 0.0001.

A Seahorse XF 24 extracellular flow analyzer was utilized to determine mitochondrial function. In this analysis, the reserve mitochondrial respiratory capacity was determined by comparing the oxygen consumption rate (OCR) before and after using the plasmin uncoupling agent FCCP with glucose as the metabolic substrate. Figure 6A depicts the variations in OCR with and without FA treatment, and Figure 6B illustrates the significant differences between groups. Since the absolute magnitude of the OCR measurements varied among experiments, we performed a normalized statistical analysis of OCR results based on protein content. FA treatment of hiPSC-CMs resulted in significantly higher ATP production and a maximum respiration rate and reserve respiration capacity compared to those in control hiPSC-CMs, indicating that FA enhanced mitochondrial respiration function.

### 2.5. AKAP1 Is Significantly Increased in Fatty Acids-Treated hiPSC-CMs

The molecular mechanism by which maturation medium (FA) promotes the maturation of hiPSC-CMs was examined by western blotting. The results suggested that the expression of the mitochondrial respiratory proteins COX5B and ATP5A and the mitochondrial β-oxidation protein PPARα and CPT1B was elevated. The expression of AKAP1 and the mitochondrial fusion proteins MFN1 and MFN2 was increased, and the expression of the mitochondrial kinetic protein p-DRP1/DRP1 was increased (Figure 6C,D), suggesting that FA promoted maturation of hiPSC-CMs may be realized through the AKAP1–DRP1 axis, which regulates mitochondrial dynamics.

### 2.6. Inhibition of AKAP1 Disrupted Mitochondrial Dynamics in hiPSC-CMs

**Figure 7:**
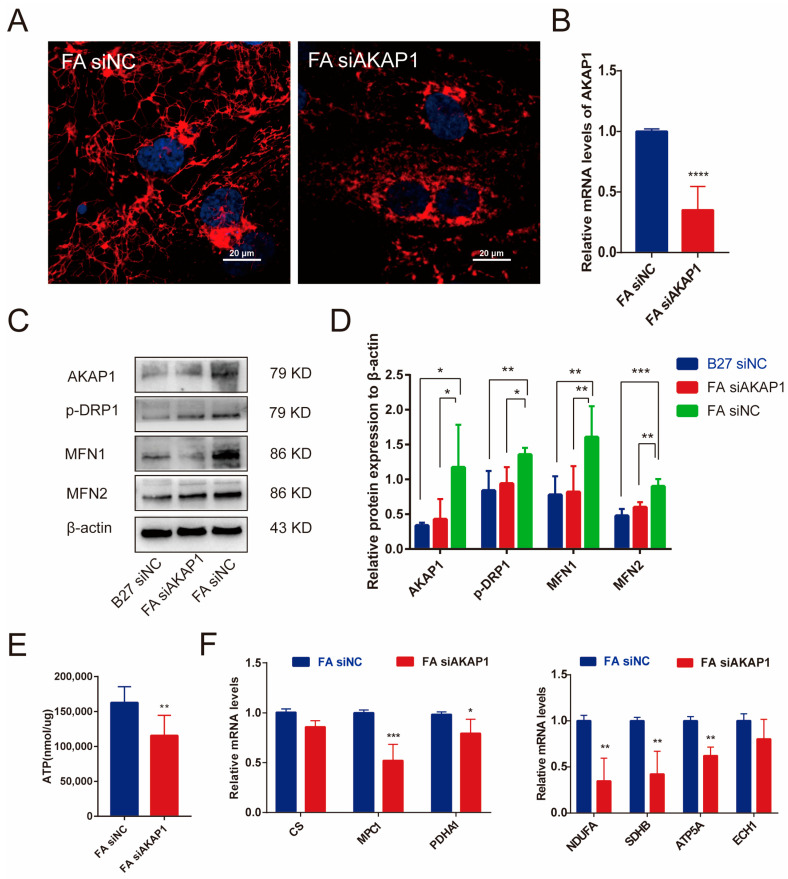
AKAP1 knockdown blocked the mitochondrial quality in hiPSC-CMs. (**A**) Mitochondrial morphology of FA siNC and FA siAKAP1 hiPSC-CMs. (**B**) Real-time PCR analysis of AKAP1 gene expression in FA siAKAP1 and FA siNC hiPSC-CMs. **** *p* ˂ 0.0001. (**C**,**D**) Western blot analysis of relative expression levels of AKAP1, p-Drp1, MFN1, and MFN2 in hiPSC-CMs between FA siAKAP1 and FA siNC hiPSC-CMs; *n* > 3, * *p* ˂ 0.05, ** *p* ˂ 0.01, *** *p* ˂ 0.001. (**E**) ATP level in hiPSC-CMs between FA siAKAP1 group and FA siNC group; *n* = 3. ** *p* ˂ 0.01. (**F**) Real-time PCR analysis of CS, MPC1, PDHA1, NDUFA, SDHB, ATP5A, and ECH1 gene expression. *n* > 3. * *p* ˂ 0.05, ** *p* ˂ 0.01, *** *p* ˂ 0.001.

To further analyze the effect of AKAP1 on hiPSC-CMs mitochondrial maturation, the morphology, and function of mitochondrial were evaluated after AKAP1 inhibition. We examined mitochondria stained with MitoTracker Red. The morphology of mitochondria was transformed from an elongated to a punctiform phenotype, illustrating the reduction in mitochondrial fusion in the AKAP1-inhibited cells compared with the siNC cells (Figure 7A). AKAP1 level in hiPSC-CMs was effectively inhibited by siRNA (Figure 7B). Western blotting further confirmed that the mitochondrial fusion proteins MFN1, MFN2, and P-DRP1 protein levels were significantly decreased in the FA siAKAP1 group compared to the FA siNC group (Figure 7C,D). Additional results showed by real-time PCR also suggested that mitochondrial respiratory complex-related genes (NDUFA2, ATP5A), oxidative phosphorylation-related genes (PDHA1, CS, MPC1, ECH1) were markedly decreased in FA siAKAP1 hiPSC-CMs. Not surprisingly, the ATP levels in FA siAKAP1 were significant decreased (Figure 7E).

## 3. Discussion

Although hiPSC-CMs can contract spontaneously, their immaturity distinguish them from adult CMs, with the former exhibiting a phenotype similar to that of fetal CMs [24,25]. Thus, promoting the maturation of hiPSC-CMs has garnered considerable interest [9,26,27]. In our study, we observed that FA addition to culture medium promoted the maturation of hiPSC-CMs in terms of cell structure, morphology, gene expression, and energy metabolism, as indicated with hiPSC-CMs cultured in FA-containing maturation medium and proteomic sequencing used to investigate the maturation mechanism of hiPSC-CMs.

The maturation of CMs is a complex process, and hallmarks of maturation are characterized by changes in cellular morphology and structure, gene expression levels, electrophysiology, and metabolism, among others features [28,29]. One of the typical characteristics of mature CMs is mitochondrial maturation [30]. In hiPSC-CMs treated with FA, cellular myofibers formed with a more elongated morphology than those in the B27 group. Correspondingly, myofiber-related protein levels were also changed. Adult CMs predominantly expressed TNNI 3, whereas fetal and neonatal CMs mainly expressed TNNI1 [31]. The switch from TNNI1 expression to TNNI3 expression suggested that FA-treated hiPSC-CMs exhibit a more mature structure. In addition, FA supplementation increased bioenergetic capacity, which was possibly maintained via active β-oxidation, and is consistent with the elevated expression of PPARα, CPT1B, and COX5B. Increased membrane potential, sustained development of a mitochondrial network, and enhanced mitochondrial respiratory function were observed in FA-treated hiPSC-CMs by immunofluorescence and a Seahorse XF24 extracellular flux analyses, suggesting adaptive mitochondrial structural and functional maturation.

In developing CMs, adaptive changes in mitochondrial bioenergetic capacity are essential for sustaining high energy output and metabolic activity [32,33,34]. In this process, mitochondrial dynamics including fusion and fission events are essential for remodeling the mitochondrial network [35]. The regulation mitochondrial dynamics has been reported to stimulate the maturation of CMs [36]. In our study, we found that the mitochondrial dynamics were altered in FA-treated hiPSC-CMs, as confirmed by increased levels of the mitochondrial fusion proteins MFN1 and MFN2, but the precise mechanisms remain incompletely understood.

The AKAP1 level was significantly elevated in FA-treated hiPSC-CMs, as determined by proteomic sequencing, the results of which were validated. In addition, AKAP1 expression was also increased when the culture time was prolonged, promoting the maturation of the hiPSC-CMs. AKAP1 is widely expressed in tissues and plays an important role in fertility, neurodegeneration, and neuroprotection, but the role of AKAP1 in CMs has remained unclear for a long time [37]. Currently, it is believed that AKAP1 anchors PKA and other binding chaperones to the cytoplasmic surface of the outer mitochondrial membrane [38,39]. AKAP1 coordinates the spatial organization and temporal regulation of cAMP and related signaling cascades in cardiac and vascular cells, contributing to cardiac physiology, including Ca2+ cycling, cardiac contractility, action potential duration, and to pathophysiological processes, such as arrhythmias, CM hypertrophy, heart failure, and hypoxia-adaptive responses [40,41,42]. In a mouse model of myocardial ischemia, AKAP1 deficiency promoted mitochondrial damage and mitochondrial reactive oxygen species(mtROS) production and enhanced myocardial mitochondrial phagocytosis and apoptosis [43].

Drp1 is a large GTPase with an N-terminal GTPase structural domain that hydrolyzes GTP to provide energy for fission. Reversible phosphorylation of Drp1 is a crucial regulatory event in mitochondrial morphology. In response to β-adrenergic stimulation of isoprenaline in the myocardium, cAMP-activated AKAP1 promoted the phosphorylation of site Ser637 in the GTPase effector domain (GED) of human Drp1, which was mediated via co-anchored PKA, which in turn inhibited the GTPase activity of Drp1 [44,45], increasing mitochondrial stability and cell survival. Consistent with literature reports, our study revealed that an increase in AKAP1 level was accompanied by phosphorylation of the Ser637 site in the Drp1 protein and the increased expression of the mitochondrial fusion proteins MFN1 and MFN2 in FA-treated hiPSC-CMs. Knocking down AKAP1 resulted in attenuated phosphorylation of Ser637 in DRP1, reduced the mitochondrial fusion rate, and decreased ATP level. These results suggest that AKAP1 may play an essential role in regulating mitochondrial dynamics during the maturation of FA-treated hiPSC-CMs.

Moreover, by performing proteomic sequencing, we found that cytochrome c oxidase (COX17) expression was significantly elevated in FA-treated mature hiPSC-CMs. The AKAP1 complex can phosphorylate the mitochondrial substrate COX, the terminal enzyme of the respiratory chain that controls the rate-limiting step of mitochondrial respiration. By binding tyrosine phosphatase D1 (PTPD1), recruiting the tyrosine kinase Src to mitochondria, and activating cytochrome c oxidase (COX), AKAP1 increased the oxidative phosphorylation and ATP synthesis rates [43,45].

In conclusion, we demonstrate that by actively metabolizing FA as the primary energy-related substrate, hiPSC-CMs maintain an extensive filamentous mitochondrial network and show increased mitochondrial respiratory function, elevated mitochondrial membrane potential and enhanced ATP levels, leading to an advanced developmental phenotype. The promotion of a mature phenotype in hiPSC-CMs induced by FA metabolic substrates may have been due to AKAP1 promoting the phosphorylation of Drp1Ser637, which regulates mitochondrial dynamics. This experiment lacked a direct comparison with human heart tissue; therefore, a precise determination of the CM stage on postnatal day 30 after FA supplementation remains unclear. Nevertheless, this study lays the groundwork for a better understanding of ultrastructural adaptations and cardiac bioenergetics in hiPSC-CMs. In addition, this study contributes to the advancement of cardiac physiology research pertaining to mitochondrial development and metabolic adaptations, thereby offering a fresh perspective on myocardial regeneration, disease modeling, and drug evaluation.

## 4. Materials and Methods

### 4.1. Generation and Characterization of hiPSC-CMs

Undifferentiated urine-derived hiPSCs from CELLAPY Biotechnology (Beijing, China) were planted on Matrigel-coated 6-well cell plates and cultivated in PSCeasy medium (CELLAPY Biotechnology, Beijing, China). When hiPSCs reached 90% confluence, cells were passaged with digest (CELLAPY Biotechnology, Beijing, China) and plated on Matrigel-coated 12-well cell plates. At 95% confluence, hiPSCs were replaced with Roswell Park Memorial Institute (RPMI, Buffalo, NY, USA) 1640 with B27 (insulin-free) medium supplemented with 6 μM GSK3- inhibitor CHIR99021 (CHIR) (Selleck, Washington, DC, USA) for 2 days. On day 3, 5 μM IWP2 (Selleck, Washington, DC, USA) was introduced to insulin-free RPMI/B27 media. On day 5, this medium was replaced for two days with an insulin-free RPMI/B27 medium. Then, on days 7–13, culture with RPMI/B27 medium. In addition, on day 13, cardiomyocytes were cultivated for three days in glucose-free RPMI/B27 media supplemented with 4 mM L-lactate sodium (Sigma, St. Louis, MI, USA) for purification. On day 18, cells were dissociated with TrypLE (Gibco, Grand Island, NY, USA) and planted with RPMI/B27 medium in another Matrigel-coated 12-well plate. On days 23–30, a maturation medium (fatty acids in glucose-free RPMI/B27 media) was supplied, and the medium was changed daily. All cultures were developed at 37 °C with 5% oxygen and 5% carbon dioxide.

### 4.2. Fatty Acid-Albumin Compound

Quantities of 10 mM palmitic acid, 20 mM sodium oleate, and 6 mM sodium linoleate were obtained from Kuntron Reagent Company (Beijing, China). The total concentration of fatty acids in neonates is approximately 300 μM, with palmitic acid comprising roughly 34%, oleic acid approximately 27%, and linoleic acid 15% [13]. HiPSC-CMs were therefore treated with equal proportions of linoleic acid albumin, oleic acid albumin, and palmitic acid albumin to imitate the fatty acid-rich environment in vivo. We supplied 115.5 M fatty acids (including 52.5 M palmitic acid albumin, 40.5 M oleic acid albumin, and 22.5 M linoleic acid albumin) to the RPMI (glucose-free)/B27 medium based on research indicating that one million cells use approximately 57 ± 7 mM fatty acids in two days [13]. On day 23, hiPSC-CMs were treated with 115.5 M FA in RPMI (glucose-free)/B27 medium and incubated continuously for seven days.

### 4.3. Proteomics Sequencing and Results Analysis

HiPSC-CMs treated with or without fatty acids (*n* = 3 in each group) were collected for proteomics analysis. Briefly, samples were placed in lysis buffer and sonicated on ice, then centrifuged at 12,000 rpm at 4 °C for 10 min, and the supernatant was collected to determine the protein concentration. TCA was added to precipitate the proteins, and then the precipitate was collected by centrifugation at 4500× *g* for 5 min. The precipitated proteins were washed 3 times with acetone. The protein sample was then redissolved in 100 mM TEAB and ultrasonically dispersed. Trypsin was added at a 1:50 trypsin-to-protein mass ratio for the first digestion overnight. The sample was reduced with 5 mM dithiothreitol for 30 min at 56 °C and alkylated with 11 mM iodoacetamide for 15 min at room temperature in darkness. Finally, the peptides were desalted by C18 SPE column. The peptides were dissolved with liquid chromatography mobile phase A and then separated using a NanoElute ultra high performance liquid phase system. The peptides were separated by the UHPLC system and injected into the capillary ion source for ionization and then analyzed by timsTOF Pro mass spectrometry.

### 4.4. Transmission Electron Microscope

After washing with D-PBS, cells were collected and fixed with 2.5% glutaraldehyde solution for 24 h. The fixed cells were embedded in epoxy resin following dehydration using a gradient of ethanol and methanol. On sectioned samples, uranyl acetate and citrate staining were then performed. Through the application of transmission electron microscopy (TEM; H-7500), TEM pictures were acquired.

### 4.5. Real-Time Quantitative PCR and RNA Extraction

Total RNA was extracted with RNAiso Reagent (TaKaRa) according to the instructions. cDNA was generated by reverse transcription of RNA samples using the PrimeScriptTM RT kit with gDNA Eraser (TaKaRa). TB Green Premix Ex Taq kit (TaKaRa) was used to measure mRNA expression. mRNA levels were normalized to beta-actin mRNA levels. Primer sequences are listed in Table S1, and all primers were synthesized by Shanghai Bioengineering Technology Co., Shanghai, China.

### 4.6. Immunofluorescence Staining

Cultured cells were fixed with 4% paraformaldehyde for 20 min, and then permeabilized with 0.5% Triton X-100 for 15 min. Then, they were incubated for 1 h with 5% bovine serum albumin (BSA) in PBS. The primary antibody was incubated overnight at 4 °C with samples. The following day, after washing with PBS, samples are incubated with secondary antibody at room temperature for 1 h. After washing with PBS, the cells were incubated for 20 min at room temperature with Hoechst 33,342 (Beyotime, Beijing, China). Images of Immuno-A fluorescence staining were captured with a confocal microscope (Nikon, Japan) and analyzed with NIS-Elements software 4.50.

### 4.7. Mitochondrial Morphology and Membrane Potential Analysis

The nuclei were initially stained for mitochondrial labeling. In this experiment, cells were treated with Hoechst dye, incubated for 16 min at 37 °C, and washed three times with D-PBS. The mitochondria of hiPSC-CMs were then stained for 25 min at 37° with a pre-warmed 0.2 M Mito Tracker working solution (Beyotime, China) or JC-1 solution (Beyotime, China). A confocal microscope A1R was utilized to analyze the staining and fluorescence intensity (Nikon, Tokyo, Japan). We analyze the mitochondria morphology using Mitochondria Analyzer as previously described [46,47,48]. The formula “(Perimeter^2^)/(4PiArea)” was utilized to determine the mean form factor (FF) value, which reflects both the length and degree of branching of mitochondria. Using the formula “(Major Axis)/(Minor Axis),” a mean aspect ratio (AR) value was determined for each cell as a measure of mitochondrial length. To measure mitochondrial membrane potential ((ΔΨm), the JC-1 kit was utilized. Green fluorescent monomers signify low ΔΨm for JC-1 staining, but red fluorescent aggregates indicate high ΔΨm.

### 4.8. siRNA Transfection

The human AKAP1 siRNA(RIB BIO, China) and negative control siRNA(RIB BIO, China) were used in this study. The siRNAs and Lipofectamine RNAiMAX reagent (Thermo Fisher Science, Waltham, MA, USA) were diluted with Opti-MEM reduced-serum medium (Gibco,USA). siRNA transfection was performed on day 27 of cell differentiation, and the cells were collected 48h later.

### 4.9. Measuring ATP Content

An ATP assay kit (Beyotime, China) was used to measure the amount of ATP. Briefly, the cells were lysed, centrifuged to obtain the supernatant. The reagent was then applied to the opaque 96-well plate, where the reagent mixed cell supernatant and ATP amount were detected. The RLU value was measured using a multifunction enzyme labeling instrument. The protein concentration served to normalize the ATP concentration.

### 4.10. Western Blot

Cells were added to the lysis buffer (KeyGEN, Beijing, China) containing phosphatase inhibitor and protease inhibitor, homogenized on a shaker at 4 °C for 30 min, and then centrifuged at 4 °C (12,000 rpm) for 15 min. The concentration of proteins in the supernatant was determined by utilizing the Enhanced BCA Protein Assay Kit (KeyGEN, Beijing, China). The protein samples were mixed with 5× buffer at a ratio of 4:1 and boiled them for 10 min. A total of 20 ug/lane protein was electrophoresed on polyacrylamide–SDS gels before being transferred to polyvinylidene fluoride (PVDF) membranes (Millipore, Burlington, MA, USA). The protein bands were blocked using TBST solution containing 5% BSA for 1 h at room temperature, followed by overnight incubation at 4 °C with the primary antibody. The following day, after being washed three times with TBST, protein bands were incubated with the appropriate secondary antibodies for 1 h at room temperature, and images were acquired using a ChemiDocTM Touch Imaging System (Bio-Rad, Hercules, CA, USA). The quantity of proteins was normalized to β-actin. Antibodies information were listed in Table S2.

### 4.11. Seahorse XF24 Metabolic Flux Analysis

Oxygen consumption rate (OCR) was measured by a Seahorse XF24 extracellular flux analyzer (Agilent Technologies, Santa Clara, CA, USA). On day 20, hiPSC-CMs were digested and subsequently plated on Seahorse XF-24 cell plates at a density of 1 × 10^5^ cells/well. After 48 h, hiPSC-CMs resumed beating and were treated with fatty acid (FA) complexes. An XF Cell Mitochondrial Stress Kit (Agilent Technologies, USA) was used to analyze mitochondrial function. One hour before the assay, cells were maintained in a non-CO^2^ incubator at 37 °C with Seahorse XF basal medium containing 1 M glucose, 100 mM pyruvate, and 200 mM L-glutamine. OCRs were measured by continuous automated injections, including oligomycin (2.5 μM), FCCP (2 μM), rotenone (0.5 μM), and antimycin A (0.5 μM). Basal respiration, maximal respiration, proton leakage, ATP production, and non-mitochondrial respiration were analyzed in an XF24 analyzer. Results were normalized to the protein per μg measured by the BCA protein assay kit.

### 4.12. Statistics

This study performed each experiment at least three times. GraphPad Prism version 7 was utilized for the statistical analysis. Data with normal Gaussian distribution were analyzed by the *t*-test and data with non-Gaussian distribution were analyzed by Mann–Whitney U-test. For multi-groups, data analysis was performed by ANOVA. *p*-values less than 0.05 were considered significant.

## Data Availability

The data used to support the findings during the study are available from the corresponding author upon reasonable request.

## Supplementary Materials

The supporting information can be downloaded at: https://www.mdpi.com/article/10.3390/ijms24098112/s1.
