# Supplementary material for: AKAP1 Regulates Mitochondrial Dynamics during the Fatty-Acid-Promoted Maturation of Human-Induced Pluripotent Stem Cell-Derived Cardiomyocytes as Indicated by Proteomics Sequencing
Source: Int J Mol Sci. 2023 Apr 30;24(9):8112. doi: 10.3390/ijms24098112 (PMC10178876; doi:10.3390/ijms24098112)
Supplement: Supplementary file 1 [file ijms-24-08112-s001.zip › Supplementary materials.pdf]

	Forward（5'to3')	Reverse（5'to3')
MYH7	GAGGACAAGGTCAACACCCT	CGCACCTTCTTCTCTTGCTC
MYL3	TCACACCTGAGCAGATTGAAGA	GCTGGAGCATAGGCAGGAAAG
TNNI1	CAGCTCCACGAGGACTGAAC	CTCTTCAGCAAGAGTTTGCG
TNNI3	CCTCAAGCAGGTGAAGAAGG	CAGTAGGCAGGAAGGCTCAG
TNNT2	AGCATCTATAACTTGGAGGCAGAG	TGGAGACTTTCTGCTTATCGTTG
ACOX1	CACAAGTAAACCAGCGTGTAAA	GTTCTTAGCCCACTCAAACAAG
CACNA1C	AGTCTCCACCCGCCACCAAG	GCTCCTCCTCATCCTCTTCTCCTG
CPT1B	CGCCGTAAACTGGACCGTGAAG	CCGAGTGGTGTTGAACATCCTCTC
KCNJ4	CTCTTCTGGTGTATCGCCTTCT	CACGCACCGGAACCCATAG
PPARα	TCGGCGAGGATAGTTCTGGAAGC	ACCACAGGATAAGTCACCGAGGAG
RYR2	ACAACAGAAGCTATGCTTGGC	GAGGAGTGTTCGATGACCACC
MFN1	TGAGGCAGTTTGGCATCTGT	CGCCTTCTTAGCCAGCACAA
MFN2	CAATCTGAGGCGACTGGTGA	GTCCTGACTTCACCTTCCCG
DRP1	TGCTTCCCAGAGGTACTGGA	CCATGTAGCAGGGTCATTTTCT
AKAP1	AGGTGGAGGCTGGTGCTGTG	TTCTGGAGGCTCTGTGACACTGG

Table S1 Primer sequences for Quantitative Real-time PCR.

**Antibodies**	**Manufacturer**	**Assay**	**Dilution**
Anti-SOX2	Proteintech	IF	1:200
Anti-OCT4	Proteintech	IF	1:200
Anti-cTnT	Proteintech	IF	1:200
Anti-cTnI	Proteintech	IF	1:200
Anti-α-actinin	Proteintech	IF	1:200
Anti-Nanog	Proteintech	IF	1:200
Alexa Fluor488 anti-rabbit IgG	Beyotime	IF	1:500
CY3 anti-rabbit IgG	Beyotime	IF	1:500
Anti-ATP5A	Proteintech	WB	1:1000
Anti-COX5B	Proteintech	WB	1:1000
WB	Proteintech	WB	1:1000
Anti-Phospho-DRP1(ser637)	Affinity	WB	1:1000
Anti-AKAP1	Proteintech	WB	1:1000
Anti-MFN1	Proteintech	WB	1:1000
Anti-MFN2	Proteintech	WB	1:1000
Anti-PPARα	Affinity	WB	1:1000
Anti-CPT1B	Proteintech	WB	1:1000
Anti-β-actin	ZEN-BIOSCIENCE	WB	1:5000
Anti-rabbit IgG	Proteintech	WB	1:5000
Anti-mouse IgG	Proteintech	WB	1:5000

Table S2 Antibodies information
